# Cavidad idiopática de Stafne: características y consideraciones imagenológicas. Una revisión

**DOI:** 10.21142/2523-2754-0903-2021-076

**Published:** 2021-10-06

**Authors:** Alexandra Gabriela Cruces Valdivia, Gustavo Adolfo Fiori-Chíncaro, Ana María Agudelo-Botero

**Affiliations:** 1 Facultad de Estomatología, Universidad Inca Garcilaso de la Vega. Lima, Perú. ale_cv90@hotmail.com Universidad Inca Garcilaso de la Vega Facultad de Estomatología Universidad Inca Garcilaso de la Vega Lima Peru ale_cv90@hotmail.com; 2 Instituto Latinoamericano de Altos Estudios en Estomatología (ILAE). Lima, Perú. gfiori@ilaeperu.com Instituto Latinoamericano de Altos Estudios en Estomatología (ILAE) Lima Perú gfiori@ilaeperu.com; 3 Facultad de Estomatología, Universidad Militar Nueva Granada, Fundación Universitaria CIEO. Bogotá, Colombia. ortoamariabotero@gmail.com Universidad Militar Nueva Granada Facultad de Estomatología Universidad Militar Nueva Granada Fundación Universitaria CIEO Bogotá Colombia ortoamariabotero@gmail.com

**Keywords:** Stafne, cavidad idiopática de Stafne, cavidad ósea de Stafne, cavidad ósea, Stafne, idiopathic Stafne cavity, Stafne bone cavity, bone cavity

## Abstract

En 1942, el Dr. Edward Stafne presentó una serie de casos con características radiográficas de imágenes radiolúcidas con forma redondeada y bien delimitadas que asemejaban patologías maxilares, que tenían algunos aspectos en común como la ubicación cercana al ángulo mandibular y la relación con la base mandibular, a los que se refirió como defecto óseo. A este defecto se le conocería más adelante con diferentes nombres.

Clínicamente es asintomática, no suele palparse intraoralmente ni presentar signos extraorales. Por ello, en la mayoría de los casos será un hallazgo radiológico accidental, el cual se presenta como una imagen radiolúcida delimitada, elíptica o redondeada, su diámetro varía de 1 a 3 centímetros, aproximadamente, y está delimitada por una osteocondensación en sus límites anteroinferiores. Según su ubicación, es clasificada en tres variantes; anterior, posterior y de rama.

Se debe considerar esta entidad como una variante de normalidad y asociarla con diagnósticos alternativos. Por ello, esta revisión brinda información sobre su historia y sus características generales y radiográficas, con el fin de contribuir a una mejor visión respecto de este tipo de hallazgos.

## Abstract (en)

In 1942, Dr. Edward Stafne presented 35 cases of asymptomatic, clearly defined, round or ovoid radiolucencies occurring near the angle of the mandible, with a greater incidence below the lower dental canal, between the mandibular angle and the roots of the first lower molar, which he referred to as a bone defect. This bone defect later became known by other names.

Clinically, Stafne’s idiopathic bone cavity (SIBC) is asymptomatic, is usually not palpated intraorally and has no present extraoral signs. Thus, in most cases it is an accidental radiological finding, which appears as a delimited, elliptical or rounded radiolucent image, with a diameter ranging from 1 to 3 cm, and delimited by osteocondensation in the anteroinferior limits. According to the location of the findings, they are classified as; anterior, posterior and branch.

This entity should be considered as a variant of normality requiring differential diagnosis to achieve an accurate diagnosis. The present review provides information on the history, and general and radiographic characteristics of SIBC to facilitate diagnosis when presented with this type of findings.

## INTRODUCCIÓN

El Dr. Edward Stafne, en 1942, presentó 35 casos de radiolucidez asintomática bien delimitada, redonda u ovoide, los cuales se presentaban cerca del ángulo de la mandíbula, con mayor incidencia debajo del conducto dentario inferior, entre el ángulo mandibular y las raíces del primer molar inferior, a las que se refirió como defecto óseo ^2^^-^^4^. A este defecto óseo se le conocería más adelante con diferentes nombres, como cavidad ósea de Stafne, quiste óseo de Stafne, quiste estático, cavidad ósea mandibular, cavidad ósea lingual, concavidad ósea idiopática de la mandíbula, inclusión mandibular de la glándula salival, defecto estático, cavidad ósea latente, cavidad idiopática de hueso, defectos mandibulares de cortical lingual, entre otros ^5^^,^^7^^,^^17^.

Clínicamente, la lesión es asintomática, no suele palparse intraoralmente ni presentar signos extraorales ^6^^,^^7^^,^^15^; por ello, en la mayoría de los casos, es un hallazgo radiológico accidental. Se presenta como una imagen radiolúcida delimitada, elíptica o redondeada, con un diámetro que varía entre 1 y 3 centímetros, aproximadamente, delimitada por una osteocondensación en sus límites anteroinferiores ^7^^,^^13^^,^^14^.

Según su ubicación, es clasificada en tres variantes: anterior, posterior y de rama. La variante anterior se encuentra en el área de la glándula sublingual, la región anterior o el cuerpo de la mandíbula. La variante posterior se encuentra en la zona de la glándula submandibular, que está en la región posterior de la mandíbula. La variante de rama se relaciona con el área de la glándula parótida ^30^^,^^31^. Hay informes en la literatura en los que la cavidad ósea de Stafne tiene variantes que no toman en cuenta dicha clasificación y se presentan en la región anterior de la mandíbula, la rama ascendente de la mandíbula, la región subcondílea, el cuello de la mandíbula, casos simultáneos anterior y posterior, etc. Además, se encuentran casos en los que la cavidad no es única, sino que también puede ser bilobulada, multilocular (presentar varias cavidades) o presentar expansión bucal ^6^^,^^12^^,^^24^^,^^17^.

A lo largo del tiempo, se consideraron varias patologías para el diagnóstico diferencial: quiste radicular como quiste óseo traumático, ameloblastoma, quiste periapical, queratoquiste ontogénico, quiste óseo simple, lesión central de células gigantes, displasia fibrosa, angioma, mieloma y lesiones fibro-óseas ^2^^,^^11^^,^^25^. Para llegar al diagnóstico tenemos la radiografía panorámica, la cual ofrece amplia información. En los casos en los que no se puede dar un diagnóstico definitivo, se utilizan estudios auxiliares como la tomografía computarizada, la resonancia magnética y la sialografía de glándula submandibular ^2^^,^^8^^,^^15^.

Por lo expuesto, se debe tomar muy en consideración el defecto óseo de Stafne, tenerlo como referente para proporcionar un diagnóstico certero y así evitar intervenciones innecesarias. Esta revisión de la literatura brindará información sobre su historia, frecuencia, incidencia, características generales y radiográficas, lo que ayudará a lograr una mejor visión con respecto a este tipo de hallazgos.

## MATERIALES Y MÉTODOS

Para la elaboración de este artículo, se llevó a cabo una revisión literaria sistematizada de artículos referentes al tema publicados hasta enero 2021, mediante las bases de datos Medline vía PubMed, SciELO y Scopus. También se consultaron diferentes revistas científicas, como *Oral Surgery*, *Oral Medicine*, *Oral Pathology and Oral Radiology*; *Endodontics Journal*, *Journal Oral Maxillofacial Pathology*, *International Endodontic Journal*, *Oral Radiology e Iranian Endodontic Journal*. Las palabras claves utilizadas para esta investigación fueron: “Stafne” y “Defecto óseo de Stafne”.

### Evolución histórica

El defecto óseo que lleva el nombre de Edward Stafne fue descrito por primera vez en 1936, por Louie Austin, quien lo describió como un quiste, el cual no crecía más allá de ciertos límites, debido a que ocurría un drenaje interno que lo encapsulaba y esto hacía que no traspase las superficies óseas; por ello, lo calificó como una patología constante o intermitente ^14^.

Posteriormente, el Dr. Edward Stafne, que laboraba en el Departamento de Odontología y Cirugía Oral de la Escuela de Posgrado de Medicina de Mayo, en Rochester (Minnesota, EE. UU.), el 1 de noviembre de 1942, informó sobre 35 casos accidentales con una misma apariencia radiográfica y ubicación, lo cual le hacía pensar que era un factor etiológico común ^1^^,^^31^^,^^33^. Las 35 cavidades se encontraron en 34 pacientes, 17 en el lado derecho y 18 en el izquierdo, en veintiocho hombres y seis mujeres con una edad promedio de 53 años. Los hallazgos se realizaron en películas intraorales y, en algunos casos, extraorales de mandíbula lateral ^1^. 

Las cavidades variaban de 1 a 3 cm y se encontraban debajo del canal mandibular, delante del ángulo mandibular del tercer molar, y estaban limitadas por hueso esponjoso, y las de mayor tamaño se expandían hasta la superficie del hueso, con una discontinuidad en el borde inferior de la mandíbula, clínicamente se podía sentir mediante palpación. Con respecto a sus características radiográficas, se describe una cavidad de forma circular u ovaladas, las paredes eran densas y gruesas, diferentes de las producidas por quistes de origen dental revestidas por epitelio. La literatura refiere que Stafne siguió cinco de los defectos durante 5 a 11 años, sin notar cambio alguno en tamaño o características ^1^.

Más adelante, en 1955, Jacobs realizó la primera exploración quirúrgica del defecto óseo de Stafne, en la que encontró una parte de la glándula submaxilar ^8^. En 1957 Euclid, Richard y Zikind publican el primer reporte de defecto óseo de Stafne en la zona anterior de un hombre blanco de 46 años. Mediante el examen radiográfico, se encontró una imagen compatible con quiste en la región del canino y el premolar inferior izquierdos, con borde esclerótico bien definido. De acuerdo con el examen clínico, ambos dientes eran vitales. Se realizó la extracción de la lesión el 9 de agosto de 1955 y se observó que el hueso era normal en color y estructura, pero tenía la placa vestibular adelgazada, el tejido se movía libremente, solo había un pequeño pedículo adherido en lingual y el defecto encontrado medía entre 2 y 3 mm de diámetro. El examen microscópico reveló una mezcla de estructura glandular compuesta de mucosa y serosa que se asemejaba a los conductos excretores de la glándula salival sublingual. El 9 abril de 1956, se mostró una evidencia de reosificación del área de la lesión ^41^.

### Teorías del origen, epidemiología y clasificación

La etiología y la patogenia de la depresión o cavidad siguen siendo poco conocidas. La primera teoría defendida por Stafne fue que una porción de la glándula salival es atrapada durante el desarrollo y la osificación de la mandíbula ^32^. A lo largo del tiempo se han descritos defectos relacionados con las glándulas sublingual y parótida, con distintas localizaciones, y en algunos casos los autores aplican el termino quiste óseo de Stafne a las lesiones de cualquiera de las glándulas salivales ^11^.

Existen diversas teorías para explicar su origen del defecto óseo de Stafne. La primera la considera un defecto congénito que ocurre durante el desarrollo, cuando la glándula submandibular queda atrapada en la parte posterior de la mandíbula. Stafne planteó que podría ser una malformación ocasionada durante el desarrollo fetal; otros autores señalan que podría deberse a la captura de una porción glandular y la osificación mandibular. Seward dice que lo que mantiene esta hipótesis es la frecuencia en la posición, la similitud en su apariencia, la aparición rara bilateral y los pocos cambios que presenta. La principal discrepancia con esta teoría señala que el defecto es más frecuente en adultos que en niños, lo que demostraría que el desarrollo es más tardío después de la osificación de la mandíbula. A favor de esta teoría se han visto casos en los que su desarrollo es lento ^18^^-^^20^. 

La segunda apunta a la presión de la glándula salival adyacente a la superficie interna de la mandíbula. Esta teoría señala que la condición de este defecto proviene de la presión generada por hiperplasia, hipertrofia o desplazamiento de las glándulas salivales hacia la superficie del hueso adyacente cuando están en procesos de formación, lo que ocasiona una remodelación ósea como respuesta. Esto explicaría la presencia del defecto en la zona anterior relacionada con la glándula sublingual, las variables del ángulo mandibular relacionada con la glándula submandibular y las de rama ascendente asociadas con las parótidas; sin embargo, esta teoría tampoco daría respuesta de la presencia del defecto en lugares poco comunes ^18^^,^^23^^,^^24^^,^^39^.

La tercera se relaciona con el tejido blando. En algunos casos, al interior de estas cavidades se encuentra tejido blando que corresponde a vasos, nervios, tejido linfático, grasa y músculos, lo que se debe a la relación entre las estructuras adyacentes a la mandíbula ^8^^,^^18^. La cuarta es la propuesta por Lello y Makek, quienes plantean que las cavidades se producen por la isquemia que ejerce la arteria facial en su trayecto por esta área ^8^^,^^18^. 

Ninguna de las teorías expuestas ha sido validada, pero las más aceptadas son las dos primeras, y la explicación es que la primera teoría refiere que el defecto es congénito, es decir, la glándula salival es retenida durante el desarrollo y la osificación. La principal observación es que los defectos se presentan más en adultos que en niños, lo que insinúa que el defecto ocurre después de la osificación; esto con base en casos en los que la cavidad no se presentaba en radiografías panorámicas preliminares. Según Philipsen, esto se justifica porque, con el avance de la edad, las glándulas salivales mayores, y en especial la submandibular, son lugares de infiltración inflamatoria inespecífica (linfocítica) con fibrosis, hipertrofia e hiperplasia, con una intensidad variable. Este proceso varió la consistencia de las glándulas de un tejido normal a uno fibroso. En la edad mediana, la fuerza realizada sobre las estructuras puede ser capaz de ocasionar la reabsorción ósea ^26^. La segunda teoría aceptada es la que se produce por la presión de la glándula en la superficie adyacente ^(28, 29)^.

Según su ubicación, se clasifica como: a) Anterior: área de la glándula sublingual, cuerpo de la mandíbula o zona anterior; b) Posterior: zona de glándula submandibular, zona posterior de la mandíbula; c) Rama: área de la glándula parótida en la cual encontramos una variante interna y externa, en algunos casos pueden presentarse en zonas inusuales como en la rama medial, lingual anterior, bilateral, etc. ^8^^,^^25^^,^^26^^,^^31^.

Ariji *et al*., en1993, presentaron una clasificación relacionada con la cortical y el contenido. Respecto de la cortical, mencionó tres tipos: Tipo I: se presenta en la cortical lingual; Tipo II: la encontramos en vestibular y lingual de la cortical; Tipo III: agrandamiento de cortical lingual y vestibular ^5^^,^^8^^,^^35^. Con relación al contenido, señala otros tres tipos: a) Tipo G: presencia de tejido glandular; b) Tipo F: presencia tejido adiposo; c) Tipo S: Contiene tejido vascular y muscular linfático ^35^^,^^36^. 

Chaudry, en 2020, menciona limitaciones en la clasificación de Ariji *et al*. respecto de la cortical, es por eso que presenta una modificación en el sistema existente: Tipo I: la profundidad de la cavidad se limita a la porción medular de la mandíbula; Tipo II: la profundidad de la cavidad llega a la corteza bucal de la mandíbula, pero no provoca su expansión; Tipo IIa: la profundidad de la cavidad llega a la corteza bucal de la mandíbula y provoca su erosión; Tipo IIb: la profundidad de la cavidad llega a la corteza bucal de la mandíbula y provoca su perforación; Tipo III: la profundidad de la cavidad llega a la corteza bucal de la mandíbula y provoca su expansión; Tipo IV: la profundidad de la cavidad llega a la corteza bucal y provoca su expansión y perforación. El autor sugiere que esta modificación es más completa e incorpora todos los TCE linguales; por tanto, ayuda a un mejor seguimiento de estas lesiones ^35^^,^^36^. 

Liu *et al*., por su parte, consideran una clasificación adicional para cuando se emplea la tomografía computarizada de haz cónico, la cual toma en cuenta la cavidad en relación con el conducto mandibular: a) Separado: cavidad que no contacta dicho conducto; b) Contacto: cuando contacta el conducto mandibular; y c) Extendido: la cavidad se extiende por el conducto. También categoriza la relación con respecto a la cortical inferior de la mandíbula en las mismas categorías. Para los casos atípicos, Minowa et al. consideran una clasificación relacionada con las vistas axiales de la tomografía: tipo amplio, tipo estrecho, tipo “margen liso” y tipo “margen irregular” ^34^^,^^36^^,^^38^.

Epidemiológicamente la probabilidad de encontrar accidentalmente una cavidad ósea estática en una radiografía panorámica es del 0,08%, y se hallan más en hombres que en mujeres (proporción de 6 a 1), en edades entre los 50 y 70 años, que son las de mayores hallazgos. Sin embargo, se han presentado casos aislados de pacientes pediátricos y en personas adultas mayores de 70. La variante lingual posterior se ve con mayor frecuencia en la fosa de la glándula submandibular, con una incidencia del 0,10% y el 0,48%. La variante más rara es la de la glándula parótida, que se detalló por primera vez en 1985 y es también llamada variante de rama ^27^^,^^28^^,^^30^^,^^32^^,^^34^ (Tabla 1).

**Tabla 1:** Distribución de características de presentación del defecto óseo de Stafne

	NÚMERO	%
SEXO		
Masculino	79	80%
Femenino	19	20%
EDAD (*)		
Masculino	53	
Femenino	51	
LADO		
Derecha		38%
Izquierda		54%
Bilateral		7%
LOCALIZACIÓN		
Anterior		30%
Posterior		65%
Rama		5%
FORMA		
Redondeada		12%
Ovalada		87%
Multilocular		1%

(*) Promedio de edad en que fueron diagnosticados

Elaboración propia

### Aspectos radiográficos y diagnósticos diferenciales

El defecto óseo de Stafne es hallado en radiografías de rutina; en la mayoría de los casos no tiene ninguna característica clínica o de sintomatología. Es importante conocer su apariencia radiográfica para emitir un diagnóstico certero y, sea cual sea su presentación, debe ser comparada con patología de origen odontogénico o no odontogénico ^14^^,^^31^.

La apariencia común del defecto óseo de Stafne en radiografía panorámica es la ubicación de una zona radiolúcida -ya sea anterior, posterior o de rama mandibular-, con márgenes bien definidos, de forma ovalada o redonda, y puede ser unilocular o multilocular ^26^. La más común será una cavidad unilocular definida, la cual está por debajo del canal del nervio dentario inferior o cerca de los ápices de los primeros molares inferiores, con bordes bien definidos y un contenido homogéneo ^14^^,^^26^.

Minowa *et al*., en 2003, clasificaron la forma de los márgenes de la cortical del defecto óseo en cuatro tipos: amplio, estrecho, liso e irregular ^42^. Hisatomi *et al*., en 2019, llevaron a cabo un estudio sobre las características radiográficas de los defectos óseos en radiografías panorámicas, las cuales clasificaron de la siguiente manera: 1) Márgenes: según la presencia de cualquier esclerosis, fina, gruesa o sin esclerosis. Cuando está presente la clasifican como parcial (no aparece en todo el contorno del defecto) o total (aparece en todo el contorno). 2) Grado de radiolucidez interna: definida como parcialmente radiolúcida (presencia de alguna trabécula ósea dentro del defecto) o totalmente radiolúcida (no se observa nada dentro del defecto). 3) Según su forma: ovalada o redonda. 4) Relación topográfica entre el defecto y el borde mandibular: continuidad del defecto hasta la base de la mandíbula o este no toca la base ni la corteza mandibular. 5) Localización del defecto: según su proximidad a los dientes, a excepción de la variante de rama. 6) Locularidad: unilocular o multilocular. 7) Solo para la variante posterior: a) bajo la pared inferior del canal mandibular; b) bajo de la pared superior del canal mandibular y continuo a la pared inferior del canal mandibular; c) debajo de la pared superior del canal mandibular y contiguo a la pared inferior; d) debajo de la pared superior del canal mandibular y superpuesto a la pared inferior del canal mandibular; e) superpuesto a la pared superior e inferior del canal mandibular; f) continuo con la pared superior del canal mandibular; g) contiguo a la pared del canal mandibular y h) por encima del canal mandibular ^32^.

La primera opción para estudiar este defecto óseo será la radiografía panorámica, por ser uno de los primeros estudios auxiliares de rutina y que, por sus características, nos puede ayudar para establecer un diagnóstico. El siguiente examen para tomar en cuenta es la resonancia magnética, que brinda mejor resolución al observar tejidos blandos y tiene ventajas de planos múltiples, además de no exponer demasiado al paciente a la radiación ionizante ^32^. 

La TC es usada como examen auxiliar para dar un diagnóstico certero, ya que sus cortes nos muestra la forma de la cavidad y todas las características en diferentes planos. Existen estudios que afirman que las tomografías computarizadas deben estar respaldadas por resonancias magnéticas, que constituyen un método diagnostico no invasivo, debido a que no emplean radiación ionizante y son usadas en los casos del defecto óseo de Stafne para caracterizar el tejido presente en el defecto sin utilizar medios de contraste ^31^^,^^32^^,^^34^.

Otro método diagnóstico es la sialografía, la cual permite evaluar si hay presencia de tejido glandular en la cavidad, pero es un método invasivo para los pacientes y tedioso debido a los múltiples conductos de la glándula ^37^. 

El diagnóstico diferencial para el defecto óseo de Stafne se puede dividir según su formación: neurogenética, histiocítica, vascular o glandular, y resulta de gran ayuda para identificar si se trata de otra patología ^14^.

## CONCLUSIONES

La cavidad idiopática de Stafne es una condición inofensiva, asintomática, que se encuentra accidentalmente en una radiografía tomada con otro fin diagnóstico. La variante más común es la posterior, que se encuentra por debajo del conducto dentario inferior, entre el ángulo mandibular y las raíces del primer molar inferior, y que suele ser unilocular. Estas características específicas nos permiten establecer un diagnóstico certero sin necesidad de exámenes auxiliares o cirugías exploratorias innecesarias; sin embargo, cuando el aspecto radiográfico de la lesión muestra características diferentes, como imágenes múltiples, multilobuladas y variantes inusuales, deben realizarse exámenes adicionales que ayuden a confirmar o descartar el diagnóstico.

La variante anterior es un hallazgo atípico que puede presentarse en la zona anterior mandibular, así como la variante de rama mandibular. Por eso, es importante que los clínicos conozcan esta variante de la normalidad, así como los protocolos de diagnóstico y seguimiento de estos casos.

## Fuente de financiamiento:

Autofinanciado
